# Supplementary material for: Stress-Responsive Protein IFRD1 Protects Assembled Ribosomes via a Ribosome-Salvaging Mechanism
Source: bioRxiv. 2026 May 7:2026.05.03.720925. Preprint. [Version 1] doi: 10.64898/2026.05.03.720925 (PMC13174501; doi:10.64898/2026.05.03.720925)
Supplement: Supplement 1 [file media-1.pdf]

IFRD1_IP_3	IFRD1_IP_2	GFP_IP
YBX1	YBX1	IFRD1
RPL26L1	NONO	RPLP2
RPL31	SFPQ	RPL29
G3BP1	G3BP1	NPM1
RBMXL1	PABPC3	RPS11
RPS10	PABPC1	RPS3A
RPL26	RPS15A	RPL3
SRSF1	HNRNPA2E	RPL14
H2BC3	RPS10	RPL13
RPS9	SRSF1	RPL5
SRSF3	DDX6	RPL12
RPL15	RPL26L1	BCLAF1
DYNLL1	ERH	RPL10A
RPS21	RBMXL1	NCL
H1-4	RPS24	RPL26
RPL30	RBMX	RPL7A
DDX6	HNRNPK	RPLP0
NUDT21	SRSF3	RPL35
RPL37A	IFRD1	RPS6
SART1	HNRNPA3	RPL23A
RPS24	H2BC3	RPS28
PABPC1	RPL31	RPL8
SLC25A6	TIMM13	THRAP3
FUBP3	UPF1	RPS15
RPL28	NUFIP2	RRBP1
TIMM8B	ELAVL1	RPS23
RPS2	FXR2	RPL4
RELCH	MAGOH	HIST1H1C
ACTC1	RPL15	RPS14
H1-10	SRSF9	LACTB
H1-3	ACTC1	RPL28
RPL7A	RPL32	HNRNPM
RBM47	RELCH	RPL32
MSI2	G3BP2	PLXNA4
DDX3X	RPS27	RPL35A
RPL13A	RPL36A	RPL34
RPL9P8; RI	MOV10	RPL13A
RPL32	HNRNPH1	RPL21
MYCBP	DDX3X	RPL17
RPL36A	RPS8	RPL23
RACK1	DDX5	RPL11
PABPC1L	H1-4	RPS4X
TUBA1B	HNRNPM	RPL19

RPL34	LARP1	RPL7
HNRNPH1	RPS13	HIST1H1B
KHSRP	RPS20	RPS8
BCLAF1	RBM8A	DDX3X
RPL3	FUBP3	RPL17-C18orf32
LARP4	RPS14	RPS10
RPS29	RPL24	RPL22
EIF4G1	PABPC4	RPS13
SRSF9	FAM120A	RPL27
HNRNPL	EIF2S1	RPS7
LARP4B	FAM98A	RPL36AL
HMGA1	RTCB	RPS25
ILF3	RPS23	RPL27A
ZCCHC3	TIMM8B	RPL31
CEP55	SART1	RPS27
RPS15A	IGF2BP3	YBX1
DYNLL2	GDF15	RPL6
H1-5	MATR3	RPS2
WDR13	EIF2S2	TOP1
GATA6	HNRNPA1	RPL26L1
TRA2B	HP1BP3	RPL15
HNRNPH3	YBX2	RPL18A
MATR3	MAGOHB	RPL36A-HNRNPH2
SRSF7	WDR13	RPL36A
LARP1	RTRAF	DDX5
TIMM8A	MSI2	RPL37A
LSM2	ILF3	RPS18
EIF2S1	FUS	H1F0
HP1BP3	C11orf98	HNRNPUL1
SNRPB	TUBA1B	DDX17
DDX1	DDX1	SRP14
HNRNPA3	RPL9P8	RPL10
TDRD3	ATXN2L	RPL24
C11orf98	RPS2	RPL30
RPS15	SERBP1	SRSF1
RPL4	RPL26	RPL36
RBM3	EIF4E	RPS24
SRSF6	RACK1	MTDH
KIF1C	STAU2	RPS5
DAZAP1	FMR1	DHX9
PTPN3	HNRNPL	H1FX
EIF3E	RPL5	SPATS2L
HNRNPA2I	PSPC1	RPL9
IFRD1	DHX30	ATAD3B

RPLP1	ALYREF	SRP9
SNRPGP15	KHSRP	KRT37
LSM6	CAPRIN1	PLA2G2A
STRBP	BCLAF1	SSB
RPL10	RPL30	HNRNPU
RPS17	RPS5	RPS9
RPL21	RBM47	SRSF5
YBX2	H1-5	TBL2
HNRNPH2	USP10	HSPD1
PCBP3	SRSF7	ADAR
SRP9	DDX17	ILF3
ATXN2	SRSF6	PNN
CAPRIN1	SHFL	UBTF
RPL27A	EIF3E	ACTG2
MARK2	RPL18A	SNRPD2
CEP44	IGF2BP2	APOBEC3C
MTCL1	HNRNPUL1	ZC3HAV1
HNRNPA0	LARP4	ACTA2
PFDN2	HMGA1	CAPRIN1
KHDRBS1	EIF2S3	RBMX
EPS15	RPS29	MKI67
USP10	ZCCHC3	SRSF7
SNRPD2	HNRNPF	ERH
SNRPD3	TDRD3	SFPQ
HNRNPF	EIF3A	LYAR
EIF4E	SLC25A6	RACGAP1
EIF4A2	STRBP	PTBP3
PRRC2C	AGO2	EZR
CCDC9B	CEP44	GNL3
AGO3	SRP9	MOV10
TIMM13	EIF4A3	NEBL
DDX50	LARP4B	RPL18
ELAVL2	EIF3C	ZC3H18
SRPK1	RPL23	C11orf98
HBA1; HBA	GATA6	SRPK1
DDX21	TIMM8A	BAG2
PPP1CA	SYNCRIP	YTHDF1
PKP2	HNRNPH3	FAU
YTHDF2	PKP2	LRRC59
MOV10	PTPN3	YME1L1
STAU2	HNRNPU	PRPF19
ZC2HC1A	CEP55	CALML5
HNRNPU	NCBP2	KHDRBS1
CPSF6	ATP5F1C	MRPL11

DHX15	SF3B6	ALYREF
RPL36	LSM6	UHRF2
FAM120A	SLC25A4	RBM28
RPAP3	YBX3	GRWD1
RSBN1	UPF3B	TRMT1L
FMR1	NCBP1	CKAP4
SHFL	RPL7A	RBM6
UPF2	RPAP3	SAP18
PRPF6	CIRBP	TBCEL
HNRNPR	ATXN2	TRA2B
SPATS2L	EIF4G1	CDX2
HNRNPUL1	TNRC6B	PPIL2
POLR2E	PCBP3	DDX24
POLDIP3	RPL21	SERBP1
RPL5	ATP5PO	RBM10
RPS20	PRRC2C	FXR2
ADD1	EPS15	LLPH
SSB	NUDT21	AP2B1
URI1	EIF3G	ZC2HC1A
PURA	URI1	NOP2
SNRNP40	DYNLL2	RRP1B
CHTOP	DYNLL1	FAM192A
RRBP1	CASC3	ASPH
YTHDF1	DDX21	HIST1H1D
NCBP1	MTDH	KIF23
SRSF4	PFDN2	SPATS2
ASXL2	RPL6	MSN
PGAM5	PTBP1	NOP53
ZC3H18	AGO1	POLDIP3
SRSF10	PRRC2A	DHX15
RPS5	SNRNP40	DHX30
STAU1	RPS17	NVL
RBM4B	RPS11	RBM39
RBM4	RPL4	ELOA
RBMX	UXT	CNTNAP4
SERBP1	PURA	PWP1
RBM17	EIF3H	TMCO1
VAPB	YTHDF2	EXOSC10
BRK1	RPS4X	KTN1
EIF3A	LSM2	MARK2
EIF3C	HNRNPH2	RBM14-RBM4
PRRC2A	RPS9	SRPK2
RUVBL2	RUVBL2	HP1BP3
UNK	RPL10	EIF2S2

SNRPA1	EIF3B	HNRNPA1
TRA2A	HNRNPUL2	MAP7
TNRC6B	ZBTB2	C7orf50
RPS6	STAU1	RFC4
EIF2S2	C1QBP	EPRS
AKAP8	AGO3	SRP68
MACO1	SSB	PGAM5
ZBTB2	RUVBL1	OASL
RPL7	EIF3D	RNPS1
FUS	LYAR	NAT10
DHX36	ELAVL2	AP2A1
AGO2	RPLP1	ZFP36L1
HNRNPM	LSM3	NIFK
GDF15	TAF6	GLUL
DHX30	CCDC9B	YTHDF2
CCDC9	THRAP3	CCDC59
SF3B6	ZCCHC8	RIOX1
LSM8	SNRPB	AKAP8
ZC3HAV1	EIF3K	KARS
PABPC4	TUBA8	DDX52
AGO1	MRPL11	NOL7
SAFB2	ZC3HAV1	DDX27
BLTP3B	PPP1CA	NOSTRIN
SNRPA	HNRNPA0	DHX36
UPF3B	RPL3	CLASRP
CSDE1	TRA2B	SRSF6
H1-0	HNRNPR	ECT2
WDCP	EIF3I	RFC1
RSBN1L	L1RE1	GPATCH2
PPP1CC	EIF3M	MRPS23
HNRNPK	CHTOP	GTPBP4
ALYREF	SNRPD1	DKC1
PAIP1	EIF1B	ASZ1
PTBP3	MARK2	MYBBP1A
SAFB	PRPF6	PRC1
TACC2	RBM17	MTPAP
AP2A2	SNRPGP15	SDAD1
NCOA5	NDUFA4	RCC2
ASDURF	SF3B2	RBM17
TAF9B	SRRT	SRP72
ZCCHC8	POLDIP3	KRR1
EIF2S3	DDX50	SRRM2
PTPN13	EIF3L	EBNA1BP2
ZNF638	SRPK1	SRPRB

SNRPC	PPP1CC	SYNCRIP
RPL19	EPCAM	SIAH1
DNAAF10	SNRPD3	TOP2A
EIF4A3	SNU13	CDC5L
BCKDK	TAF9B	YTHDF3
CPSF7	KIF1C	NOP16
YTHDF3	SPATS2L	FXR1
TAF9	CTTN	CD3EAP
CEP170	DAZAP1	DAZAP1
OTUD4	ZC3H18	NKRF
LRRFIP1	TAF9	HMGCR
ZC3H11A	TOP3B	PRPF3
SRRT	SLC25A1	SEMA3B
PRPF8	H1-10	DDX18
FAM98A	TOP1	DDX55
CPSF4	PRKRA	EEF1E1
RPL29	RSBN1	AFF4
PHF5A	LARP1B	MARK3
TOP1	DNAAF10	EXOSC3
XRN1	ZC2HC1A	TOP2B
TAF6	RBM4	CHD1
RPL10A	LSM8	TRIM25
C1QBP	PTPN13	CCDC25
PPP1CB	ZC3H7A	RFC3
ZC3H7A	KHDRBS1	POLR1E
LARP1B	HNRNPD	DNAJB11
SRSF5	EIF4A2	DNAJC9
LYAR	MTCL1	MOGS
SLAIN2	CPSF7	HADHA
STRAP	RPL35	SF3B1
EIF3D	EWSR1	ZRANB2
KHDRBS3	ASXL2	RRP15
IGKV1-6	DDX23	SLAIN2
EIF1B	DHX15	SNRPA
RBM8A	RPL37A	DDX21
EIF3B	H1-0	GLYR1
AP2B1	ATP5MG	ASCC3
ATP5F1C	TAF10	YY1
SF3B1	SNRPE	SNU13
EIF3H	C19orf53	XRCC5
YTHDC2	ASDURF	RBM14
RFC4	CDKN2AIP	CKAP5
PUM2	YTHDF1	KNOP1
TAF8	SNRPA1	ZCCHC9

EIF4G3	TRA2A	MLLT3
PNN	NIFK	SLC16A1
RPL18A	SLC25A11	NUSAP1
CTTN	SNRPC	TUBB3
UPF1	EIF3F	CMAS
CC2D1A	RBM4B	TRIP12
YWHAZ	EFTUD2	RARS
COX5B	ADAR	CBX4
SNU13	DHX36	RFC2
GNG5	TACC2	EIF4A1
MARK3	RBFOX2	EIF2S1
MRPL11	DHX9	AIMP2
NOP16	UPF2	MELK
MTDH	HNRNPDL	STAU2
RIOX1	SAP18	MAP4
ADAR	MRPS26	CHTOP
EIF3G	SUCLG1	SRSF9
SF3B2	PHF5A	RBBP8NL
FAM83B	RPL39	MRM3
UBAP2L	RAB6A	IBTK
SUCLG1	PRPF31	OCIAD2
TFAM	TAF15	PPFIBP2
RPL18	SRSF10	PCBP1
TAF5	POLR2E	PHF8
ATP5PB	SRSF2	SRRT
ZFR	ZNF638	TJP2
TRIM29	RSBN1L	MRPS9
TAF10	AKAP8	RBBP6
MRPS23	RFC5	ACOT9
EIF3I	PPIH	LARP1
DDX23	LRRFIP1	EXOSC9
RPL35	PKP3	EFCAB7
SNRNP70	YTHDF3	SF3B6
CELF1	SAFB	CCDC9B
TOP3B	CEP170	EIF2AK2
AKAP1	SRPK2	NOP56
KCTD14	FGFBP1	ZNF146
GRSF1	IGKV1-6	RPP25
NDE1	YTHDC2	ZNF281
HNRNPUL[illegible]	XRN1	TRIM28
RPL14	PAIP1	SF1
APOBEC3[illegible]	UNK	DDX54
RBM45	BLTP3B	EPB41L5
RPL13	SURF6	NKTR

RBM7	SNRNP200	TCOF1
PKP4	CSDE1	ABCF1
RFC2	RSL1D1	HIST1H1E
YLPM1	RRBP1	HNRNPR
RPS23	MACO1	MRPS22
HELZ	PTBP3	CCDC77
LUC7L2	ILF2	KIF2A
PRRC2B	MRPS6	EXOSC5
L1RE1	PAM16	FBXL13
TAF4	HELZ	EIF2S3
TRIM25	PRPF3	MRPS7
TFB1M	PRPF4	TIGD6
PIH1D1	ATP5PB	UFM1
MRPL54	MRPS18B	MAGT1
PDRG1	ASPH	DDX60
PLA2G2A	NFX1	MATR3
TOMM22	ZFR	PAK1IP1
NOP10	NCOA5	IFI16
SUGP2	CPSF6	CLASP2
SNRNP200	CHERP	ARHGEF2
RUVBL1	SNRNP70	GNL3L
EIF3L	FIP1L1	SECISBP2
AP2M1	SUGP2	MARS
TBL2	KCTD14	KIN
SRSF2	LSM4	REPIN1
KDM1B	NDUFB4	MAP7D1
EFTUD2	YLPM1	AURKAIP1
SPOUT1	MKRN1	BCAS2
ZC3H7B	SAFB2	VTN
SLC25A11	PGAM5	QARS
SZRD1	PPP1CB	BUD13
TXNL4A	WDR11	LRRK2
NONO	DHX29	XRN2
SPATS2	SRSF5	HDGFL2
HNRNPLL	SF3B1	YTHDC2
CEP43	PKP4	CENPV
NELFE	CSNK1A1	DHX57
MRPS34	KHDRBS3	SLC29A3
CDKN2AIP	WDCP	SPTY2D1
LRPPRC	TAF5	PAF1
MARF1	RFC4	TBL3
WTAP	MRPL54	TDRD3
GTF2IRD1	PDRG1	HS2ST1
RRP1	TOMM22	SF3B3

ATP5PD	NOP10	GPATCH4
RNF41	PRPF8	CEP170
TECR	WDR33	RRS1
DNAJB6	PYM1	MRTO4
ZFP36L1	PUM2	RIOK2
UXT	EIF5	TUBA1A
UBL5	RPL22	MRPS18B
AP2A1	SRP14	MRPS35
HNRNPD	SNRPD2	EXOSC8
GTPBP4	MARK3	SKP1
BRI3BP	TAF4	WDR36
-	CPSF1	SF3B4
CHERP	CDX2	TUBA1C
CPSF1	RBM3	EXD2
RFC5	NHP2	HELZ2
SKP1	CYB5B	RBM27
CIRBP	CPSF3	MIS18BP1
MAGT1	CCDC9	RRP7A
PPIH	SPATS2	G3BP1
DKC1	RBM10	PTCD3
MRPS18B	DNAJA3	UTP18
PUM1	MRPS34	SRSF2
PRPF3	PRRC2B	PARS2
SRPK2	KRR1	U2SURP
ADD3	RRP1	NOL6
GRWD1	AP2B1	DARS
ZNF746	ZC3H11A	GRSF1
H1-2	RPS3A	MAP4K4
CIZ1	SLTM	ERI1
TAF7	SLAIN2	SDCBP
RBM42	RNF41	SCAPER
EN1	DNAJB6	ACIN1
EXOSC8	ZFP36L1	SART1
LSM5	BRK1	AATF
MED30	TAF12	NCBP1
MBD6	UBL5	VWF
NCBP3	MTREX	ELOB
HELZ2	ZC3H7B	RSL1D1
SURF6	GMPPA	NFXL1
RFC3	ZBTB5	NPM3
CKAP5	BRI3BP	SF3B2
MAP7D1	SEC13	AKAP8L
SLBP	NSUN5	CPT1A
RBM10	PURB	MTREX

DNAJA3	CC2D1A	FARP2
PRMT1	ESRP1	AHCYL1
EPS15L1	SNRNP27	RBM34
RRP1B	TARDBP	TNIK
LSM14B	RPS6	ERCC3
RPUSD4	CCDC59	EXOSC1
MRPL12	PUM1	PARP1
WDR11	PCBP2	PLRG1
SECISBP2	PRPF4B	RRP1
MKRN1	MBNL3	CFAP20
U2SURP	SF3A3	IQCE
PURB	EIF5B	RBM15
DGCR8	AP2M1	GPATCH8
PABPN1	TBL2	RSBN1
USP39	CPSF4	PES1
CPSF2	DNAJC9	SURF6
RSL1D1	SRPRB	ESRP1
CPSF3	SF3B5	KIAA1522
RPL37	LSM5	IARS
PRPF31	SF3A1	LARP4
RBMS2	NCBP3	RNF2
PUSL1	EIF4G2	DNAJA2
C7orf50	TFAM	DIDO1
SNRPE	EMD	KIF14
ATP5MF	PNN	POLRMT
PACSIN2	RFC3	RACK1
SLC25A1	PHF6	ELMSAN1
CDX2	RRS1	BCL2L2-PABPN1
NFX1	RBM7	SLTM
FXR2	TRIM25	QSOX1
RNPS1	CELF1	DHX29
SLC25A22	FAM91A1	LONP1
EPCAM	UBAP2L	FAM207A
CCAR2	MBNL1	SART3
BAP1	RPS16	KIF2C
GMPPA	AP2A2	DIMT1
KPNA2	GRSF1	ZCCHC8
EBNA1BP2	ZBTB44	RO60
NCKAP1	PIH1D1	SRPRA
MRM3	MORF4L2	DDX47
CSNK1D	PFKP	CEP170B
DLG5	NELFE	TTC3
DECR1	RBM45	NOL10
AXIN1	RPSA	CCDC137

TSPAN10	CPSF2	KIF18A
RAB11A	WTAP	ERAL1
LSM3	GAR1	ESF1
TMA16	MRPS23	AIMP1
RBBP7	C7orf50	ZNF638
FAM120C	ATP5MF	ZNF512B
WDR33	C17orf75	TRIM56
PFKL	YY1	KRT23
PFKP	RNPS1	SNRPA1
WDR5	SLC25A22	PARD3
WWP2	TAF8	MRPS31
GNL3	HELZ2	EXOSC7
DDX18	BLM	DEF6
SEC22B	BAP1	PSIP1
THOC6	BCKDK	ZNF568
LUC7L3	NUDT16L1	ZNF598
ZBTB44	SSRP1	RRP8
IGKV1-27	DKC1	AP2M1
FGFBP1	DIMT1	DDX60L
C1QC	EBNA1BP2	ZNF622
MBNL3	RBM14	UPF3B
RPL8	U2SURP	MICU1
ZNF326	RRP1B	GTSE1
DROSHA	AP2A1	SGPL1
ERI1	TECR	RBM15B
EWSR1	UQCRC2	TAF10
FAM91A1	DDX28	PCSK5
CYFIP2	TSPAN10	MYO9B
RPS19	MYCBP	TPX2
RBFOX2	DAD1	REXO1
HDAC1	FAM120C	AURKA
ABI1	AKAP1	CHSY1
KEAP1	GRWD1	EFTUD2
TNRC6A	XRN2	NUMA1
TIAL1	GNL3	DNAJC10
MBNL1	DDX18	ANKZF1
F2	SLX9	EIF3D
SPOP	MRPL24	RPN1
SOX9	TUBAL3	EML3
EXOSC6	KLHL12	CLASP1
TIMM21	MAGT1	SNRPB2
CDX1	POLB	HNRNPUL2
ATXN2L	SF3B3	SUN2
DHX29	MARF1	SETX

YTHDC1	DHX57	ALDH3A2
RPS14	USP39	CALU
BAIAP2L1	RFC2	PDCD6IP
CBX8	IGKV1-27	LARP7
TIA1	DCAF7	SCO1
PRPF4	MRM1	CDC20
FRG1	IGKV4-1	CDK13
CCDC59	MOGS	TRAM1
MRPL47	PARP2	LRBA
PHAX	TRIP4	FMNL2
TBC1D30	AGR2	MTHFSD
SF3A3	CKAP5	CBX8
ZBTB5	TAF7	INAVA
CLK3	TFB1M	SND1
EXPH5	RPS21	TSR1
SF3B3	SF1	ZC3H8
KCNQ1	EIF4G3	
DDX28	BRIX1	
C17orf75	NFIX	
YY1	OTUD4	
APOBEC3F	DDX55	
PFDN6	PACSIN2	
LSM4	COX5B	
BIN3	PNO1	
MRPS16	EXOSC6	
EXOSC3	YTHDC1	
PRSS33	PNKP	
CYB5A	CIZ1	
ZC3H13	NCKAP1	
SECISBP2L	YME1L1	
XRN2	CBX8	
SLC25A10	NSUN4	
NKAP	SIN3A	
RPL12	MUC6	
NSUN5	LRPPRC	
GSK3B	LACTB	
SYNCRIP	RBMS2	
KLHL12	PHAX	
PARP12	CLK3	
RNF213	LAMP1	
PRPF38B	PFDN6	
HBE1	EXOSC8	
EIF1AX	MRPS16	
SDCBP	EXOSC3	

HBD	MRPS15
SRSF8	MRPS17
RBM6	SPTY2D1
TRIM2	CBX4
MRPS2	SLC25A10
FIP1L1	NKRF
BYSL	LUC7L2
SLTM	KDM1B
PRPF4B	MRPS27
SAP18	EIF1AX
NIFK	CMSS1
LSM14A	PLA2G2A
ACIN1	RMI2
EIF4G2	SRSF8
SLC3A2	POP7
SF1	CHD6
METTL17	EPB41L5
NHP2	TRIM2
FAM162A	MTPAP
NTHL1	CCDC137
POLR2H	PUSL1
AP2S1	GTPBP2
MGST2	BYSL
TAF3	ST7
PATL1	RBBP4
EXD2	LSM14A
DIMT1	TDP1
RBM15	METTL17
CHD6	PIP
WASF2	OCIAD2
PRC1	SZRD1
MGST1	FAM162A
CYFIP1	ATP5F1D
SMG7	POLR2H
APC	AP2S1
EPHB3	ZNF746
RFX7	FOXK2
PAWR	TAF3
EIF3F	RNF213
DHX37	PATL1
SRBD1	EXD2
TENT2	TRIM33
NUFIP1	FUBP1
NFIA	NOP2

RARS1	SMG7
HCFC1	SART3
SNRNP27	HDAC1
CD2BP2	CBLL1
TBP	RCN2
MRPL49	PAWR
CNBP	WDR5
TWNK	WDR77
ARHGAP32	GNB1
DHX57	MAP7D1
RCN1	TOP3A
DCAF7	SRBD1
HECA	ZNF639
MUC6	NFIA
TMOD3	EPS15L1
DIS3L2	ACIN1
CPNE3	SECISBP2
EIF5B	STRAP
RPS8	THOC6
LUZP1	CD2BP2
KIF2A	MRPL49
PSIP1	CNBP
PON1	CPEB3
RBM46	PCNP
MRPS25	DDIT3
MED28	TWNK
ZMAT5	RBM27
PON3	HNRNPLL
MRPS22	TIMM50
WDR6	ERI1
SNW1	MBD6
RBBP8	AFG2B
TRIM33	WDR6
PHF6	APTX
MAP7	RCL1
SIN3A	NDUFA10
RAP1B	RIOK2
DNAJB12	RPUSD4
EXOSC1	MRPL12
NDUFB10	NSDHL
RPP25	EXOSC1
MRPS11	SSR3
RC3H1	CYFIP2
SGPL1	CPNE3

NSUN2	RBM6
GTPBP10	DGCR8
PARD3	NSUN2
MEX3D	ARMCX3
SART3	DECR1
KRR1	RPL29
MTPAP	ZNF326
PAK1IP1	NOP56
AURKAIP1	CHP1
NOP2	AURKAIP1
PPIG	IMP3
TRIM28	RPL22L1
TJP2	APOBEC3F
ATP5PO	NAP1L1
NAP1L1	FAM83G
SCAPER	FAM83B
PES1	MRPS9
FUBP1	KLHL8
FAM83G	CLDN3
EIF4B	ARL6IP1
MOGS	ARHGAP32
RALB	RMI1
FOXK2	F2
NUDT16L1	NXF1
ARL6IP1	CEP43
NAT10	DNAJC13
C5	MRM3
MKRN2	RIOX1
TOR4A	SLC25A3
TRMT10C	SRRM2
RBM22	AXIN1
RBM27	CMAS
PAN3	RAB21
CMAS	TMA16
JPH1	MRPL22
LZTS3	THOC7
RAB21	SECISBP2L
MRPS27	ZBTB25
MRPL22	GTPBP4
RAB6A	SLC25A13
THOC7	HERC2
RBM15B	KCNQ1
ZNF281	RBM42
LZTS2	SLC39A7

ZBTB25	CHMP4B
MYBBP1A	C8orf33
GFAP	EXOSC5
GPATCH4	PARD3
SLX9	THOC1
CHMP4B	LUC7L3
SYNGR2	SND1
EXOSC5	KNOP1
VPS28	EBP
KLF16	TMEM109
RBBP6	C1QC
SPTY2D1	GSTA1
ESRP1	HCFC1
THOC1	PARP12
CEMIP	SERPINC1
NKRF	KIF2A
RNF214	CPEB4
EBP	RBM15
EXOSC4	ZNF608
TAF1	DHCR7
VAPA	TRAF4
C3	EIF6
PRPF19	TMCO1
EIF6	ABI1
TMCO1	ALDH3A2
TRIM56	LTV1
AFG2B	RPL14
EPB41L5	KRI1
NFIX	ASCC3
LTV1	-
PDCD7	TENT2
POLRMT	PWP1
ZNF639	MRPL16
PNO1	MRPS7
MRPL16	CYP2S1
MRPS7	TIMM21
ZCCHC17	PFKM
KLK7	BAIAP2L1
NFIC	LEMD2
DNAJC13	KPNA2
PNKP	CYFIP1
TOE1	FRG1
YME1L1	VRK2
FLII	GATAD1

VRK2	RFX7
MFAP4	TNRC6A
TMEM70	NOP58
MTREX	NDUFS3
NOP58	RBM46
SCO2	BIN3
MED4	UBP1
TOP2B	ZNF768
CBX4	SURF4
HPSE	R3HDM4
ZNF479	MAP4
AXIN2	TAF1
CDC5L	TOP2B
KIF21A	SNW1
AGAP1	CSTF2
TENT4B	CEMIP
SMG6	TSR1
RFC1	RBM5
SPICE1	POLR2C
TIMMDC1	C3
CMSS1	KTN1
REPIN1	C5
PDZD8	AGAP1
ZNF608	RC3H1
TMEM200B	SMG6
CCDC137	PAN3
NOP56	AHR
ZNF598	PRPF38B
ZNF346	SGPL1
PPIE	NOL6
SLC25A21	TMEM45B
MAIP1	SDCBP
	REPIN1
	NMNAT1
	SSR1
	RBM15B
	MEX3D
	LAMB3
	ZNF512
	LPIN1
	MRPS2
	ZNF346
	PPIE
	NAPA

MRPL2
EPB41L4B
POLR2B
EXOSC10
