# Supplementary material for: Stress-Responsive Protein IFRD1 Protects Assembled Ribosomes via a Ribosome-Salvaging Mechanism
Source: bioRxiv. 2026 May 7:2026.05.03.720925. Preprint. [Version 1] doi: 10.64898/2026.05.03.720925 (PMC13174501; doi:10.64898/2026.05.03.720925)
Supplement: Supplement 2 [file media-2.pdf]

Supplementary Table 2. List of mouse strains used in this study

B6.129-*Bhlha15*$^{tm3(cre/ERT2)Skz}$/J	Jackson laboratories	RRID: IMSR_JAX: 029228
B6.Cg-*Gt(ROSA)26Sor*$^{tm9(CAG\text{-}tdTomato)Hze}$/J	Jackson laboratories	RRID: IMSR_JAX:007909
Nat10 flox/flox	Generated by the authors	PMID: 40081569
Ifrd1 flox/flox	RIKEN (Accession No. CDB0833K)	PMID: 26391411
Ifrd1^{tm1Lah}	Generated by Dr. Lukas Huber Medical University of Innsbruck	RRID: MGI:3043558
C57BL/6J	Jackson laboratories	RRID: IMSR_JAX:000664

Supplementary Table 3. List of short hairpin RNAs (shRNAs) and guide RNAs (gRNAs) for knockdown or knockout experiments.

Oligo	Sequence
shRNA for IFRD1_Forward	ACCGGGCAGTGGTTATAGCGATCCTTCTCGAGAAGGATCGCTATAACCACTGC
shRNA for IFRD1_Reverse	AAAAGCAGTGGTTATAGCGATCCTTCTCGAGAAGGATCGCTATAACCACTGCC
gRNA for IFRD1 KO #1_Forward	CACCGTCCTTGACGCAGATGCTTA
gRNA for IFRD1 KO #1_Reverse	AAACTAAGCATCTGCGTCAAGGAC
gRNA for IFRD1 KO #2_Forward	CACCCAATCAATATACATGCGTTC
gRNA for IFRD1 KO #2_Reverse	AAACGAACGCATGTATATTGATTG

gRNA: Guide RNA

Supplementary Table 4. List of primary antibodies used in this study

Name	Company	Species	Cat. No.	Purpose	Dilution
IFRD1	Abcam	Rabbit	ab229720	WB (Hs, Ms)<br>IP (Hs)	1:1,000<br>5μL
IFRD1	Novus	Rabbit	NBP1-87327	WB (Ms)	1:1,000
IFRD1	Proteintech	Rabbit	12939-1-AP	WB (Hs)	1:2,000
GFP	Abcam	Rabbit	Ab6556	WB	1:1,000
NAT10	Abcam	Rabbit	ab194297	WB	1:1,000
NCL	Cell Signaling	Rabbit	#14574	IF	1:200
UBTF	Cell Signaling	Rabbit	#35663	WB	1:1,000
TCOF1	Sigma	Rabbit	HPA038237	IF	1:200
RPS6	Cell Signaling	Rabbit	#2217	WB<br>IF	1:2,000<br>1:200
Phospho-S6 Ribosomal Protein (Ser240/244)	Cell Signaling	Rabbit	#5364	WB	1:2,500
4E-BP1	Cell Signaling	Rabbit	#9644	WB	1:1,000
Phospho 4E-BP1 (Thr37/46)	Cell Signaling	Rabbit	#2855	WB	1:1,000
Acetyl-CoA Carboxylase	Cell Signaling	Rabbit	#3676	WB	1:1,000
Phospho-Acetyl-CoA Carboxylase (Ser79)	Cell Signaling	Rabbit	#11818	WB	1:1,000
β-Actin	Santa Cruz	Mouse	sc-47778	WB	1:5,000
ADAR1	Cell Signaling	Rabbit	#14175	WB	1:1,000
dsRNA (J2)	Cell Signaling	Mouse	#76651	IP	5μL
dsRNA (K1)	Cell Signaling	Mouse	#28764	IP<br>IF	5μL<br>1:200
ZFP36L1	Cell Signaling	Rabbit	#30894	WB<br>IP	1:1,000<br>5uL
Ubiquitin	Santa Cruz	Mouse	sc-8017	WB	1:1,000
G3BP1	Abcam	Mouse	ab56574	IF	1:200
G3BP1	Proteintech	Rabbit	13057-2-AP	IF	1:200
LC3B	Cell Signaling	Rabbit	#43566	WB<br>IF	1:1,000<br>1:200
LC3B	Novus	Rabbit	NB100-2220	WB	1:1,000

SQSTM1	Santa Cruz	Mouse	sc-28359	WB<br>IF	1:1,000<br>1:200
RPL11	Abcam	Rabbit	ab79352	WB<br>IF<br>IHC<br>IP	1:2,000<br>1:200<br>1:200<br>2μg
RPL13	Abcam	Rabbit	ab134961	WB<br>IF	1:1,000<br>1:200
HA-tag	Cell Signaling	Rabbit	#3724	WB	1:1,000
Purified anti-HA.11 Epitope Tag	BioLegend	Mouse	#901501	IF	1:500
RPL5	Abcam	Rabbit	ab86863	WB<br>IF	1:2000<br>1:200
eIF2alpha	Cell Signaling	Rabbit	#5324	WB<br>IP	1:1,000<br>5μL
Phospho-eIF2alpha (Ser 35)	Cell Signaling	Rabbit	#3597	WB	1:1,000
RPL26	Abcam	Goat	ab157111	IF	1:200
RPL26	Cell Signaling	Rabbit	#2065	WB	1:1,000
SRP68	Abcam	Rabbit	ab157120	WB<br>IP	1:1000<br>5uL
PERK	Cell Signaling	Rabbit	#3192	WB	1:1000
SRPR	Proteintech	Rabbit	12090-1-AP	WB	1:1000
GAPDH	Cell Signaling	Rabbit	#2118	WB	1:1000
α/β-tubulin	Cell Signaling	Rabbit	#2148	WB	1:2,000
TRAPα	Santa Cruz	Mouse	sc-373916	IF	1:200
LAMP1	Cell Signaling	Rabbit	#99437	IF	1:200
Amylase	Cell Signaling	Rabbit	# 3796	IHC<br>IF	1:500<br>1:500
Puromycin	Millipore	Mouse	clone 12D10, MABE343	WB	1:2,000
TdTomato	SicGen	Goat	AB8181	IF	1:200
Ki-67	Invitrogen	Rat	14-5698-82	IF	1:500
p-Histone H3	Cell Signaling	Rabbit	#9701	IHC	1:500

Insulin	Dako	Guinea Pig	A056401-2	IF	1:500
E-cadherin	Cell Signaling	Mouse	#14472	IF	1:200
CK19	Proteintech	Rabbit	10712-1-AP	IF	1:200

WB; Western blot, IF; immunofluorescence, IHC; immunohistochemistry, IP; immunoprecipitation

Supplementary Table 5. List of qRT-PCR primers used in this study

qRT-PCR primers	Species	Sequence
IFRD1 Forward	Homo Sapiens	TGCAGTGGTTATAGCGATCCT
IFRD1 Reverse	Homo Sapiens	CCTTGTCTTCGCACTCTTATCC
Ifrd1 Forward	Mus Musculus	GTCGCATCTGTTCTTTGTATTCAG
Ifrd1 Reverse	Mus Musculus	ACAGCAAACACCAAAGCAAG
GAPDH Forward	Homo Sapiens	AAGAAGGTGGTGAAGCAGGC
GAPDH Reverse	Homo Sapiens	TCCACCACCCTGTTGCTGTA
Gapdh Forward	Mus Musculus	GGGTGTGAACCACGAGAAATA
Gapdh Reverse	Mus Musculus	AGTGATGGCATGGACTGTG
RPL5 Forward	Homo Sapiens	GGTGTGAAGGTTGGCCTGAC
RPL5 Reverse	Homo Sapiens	GGCACCTGGCTGACCATCAA
RPL11 Forward	Homo Sapiens	TCCACTGCACAGTTCGAGGG
RPL11 Reverse	Homo Sapiens	AAACCTGGCCTACCCAGCAC
RPS6 Forward	Homo Sapiens	TGGACGATGAACGCAAACTTC
RPS6 Reverse	Homo Sapiens	TTCGGACCACATAACCCTTCC
45s pre-rRNA Forward	Homo Sapiens	ACCCACCCTCGGTGAGA
45s pre-rRNA Reverse	Homo Sapiens	CAAGGCACGCCTCTCAGAT
SQSTM1 Forward #1	Homo Sapiens	GCACCCCAATGTGATCTGC
SQSTM1 Reverse #1	Homo Sapiens	CGCTACACAAGTCGTAGTCTGG
SQSTM1 Forward #2	Homo Sapiens	GACTACGACTTGTGTAGCGTC
SQSTM1 Reverse #2	Homo Sapiens	AGTGTCCGTGTTTCACCTTCC
TFEB Forward	Homo Sapiens	ACCTGTCCGAGACCTATGGG
TFEB Reverse	Homo Sapiens	CGTCCAGACGCATAATGTTGTC

Supplementary Table 6. List of plasmid constructs used in this study

IFRD1	IFRD1 (NM_001007245) Human Tagged Lenti ORF Clone	OriGENE
(N)Td-Tomato-IFRD1 (Hyperactive Piggybac Transposase)	Generated by Dr. Jeffrey W. Brown	
(C)IFRD1- HA- GFP (Hyperactive Piggybac Transposase)	Generated by Dr. Jeffrey W. Brown	
IFRD1- HA (Hyperactive Piggybac Transposase)	Generated by Dr. Jeffrey W. Brown	
hIFRD1-His (Hyperactive Piggybac Transposase)	Generated by Dr. Jeffrey W. Brown	
Transposase (Hyperactive Piggybac Transposase)	Generated by Dr. Jeffrey W. Brown	
ΔIFRD1	EIGC, Emory University, Dr. Laur Oskar	

Supplementary Table 7. List of reagents used in this study

Agar	Lamda Biotech	Cat# C110
Tris Buffered Saline, with Tween® 20, pH 8.0	Sigma-Aldrich	Cat# T9039
Triton X-100	LabChem	Cat# LC262801
PBS, 10X Sterile	Corning	Cat# 46-013-CM
Digitonin, High Purity	Sigma-Aldrich	300410-250MG
Tris Buffered Saline, with Tween® 20, pH 8.0	Sigma-Aldrich	Cat# P9039
Triton X-100	LabChem	Cat# LC262801
Tamoxifen	Toronto Research Chemicals Inc	Cat# T00600
Puromycin	Sigma	Cat# P8833
Bortezomib	Selleck Chemicals	Cat# S1013
Cycloheximide	Sigma	Cat# C7698
Harringtonine	Selleck Chemicals	Cat# S9063
Sodium (meta) arsenite,≥90%	Sigma-Aldrich	Cat# S7400-100G
Tunicamycin	Sigma-Aldrich	Cat# T7765-10MG
Thapsigargin	Selleck	Cat# S7895
Cerulein Ammonium	Bachem	Cat# 50-259-725
MG-132	Sigma-Aldrich	Cat# 474790
Rabbit Reticulocyte Lysate, Nuclease-Treated	Promega	Cat# L4960
Halt™ Protease Inhibitor Cocktail, EDTA-free (100X)	ThermoFisher	Cat # 78437
Halt™ Protease and Phosphatase Inhibitor Cocktail, EDTA-free (100X)	ThermoFisher	Cat# 78443
RIPA Lysis and Extraction Buffer	ThermoFisher	Cat# 89900
Pierce™ BCA Protein Assay Kit	ThermoFisher	Cat# 23225
2-Mercaptoethanol	Sigma-Aldrich	Cat# M3148
Dithiothreitol	Research Product International	D11000-10.0
NuPAGE™ 4 to 12%, Bis-Tris, 1.5 mm, Mini Protein Gel	ThermoFisher	Cat# NP0335
NuPAGE™ LDS Sample Buffer (4X)	ThermoFisher	Cat# NP0007
Ponceau S	Sigma-Aldrich	Cat# P7170-1L

BSA	Sigma-Aldrich	Cat# A7906
Dynabeads Protein A	ThermoFisher	Cat# 10001D
ChromoTek GFP-Trap® Magnetic Particles M-270	Proteintech	Cat# gtd
Peroxidase AffiniPure Donkey Anti-Rabbit IgG (H+L)	Jackson ImmunoResearch	Cat# 711-035-152
Peroxidase AffiniPure Donkey Anti-Mouse IgG (H+L)	Jackson ImmunoResearch	Cat# 715-035-150
SuperSignal™ West Pico PLUS Chemiluminescent Substrate	ThermoFisher	Cat# 34579
Veriblot	Abcam	Cat# ab131366
Pierce™ IP Lysis Buffer	ThermoFisher	Cat# 87787
Normal Rabbit IgG	Cell Signaling	Cat# 2729
PowerUp SYBR Green Master Mix	ThermoFisher	Cat# A25742
RNase-Free DNase Set	Qiagen	Cat# 79254
PrimeScript™ RT Reagent Kit	Takara Bio Inc	Cat# RR037B
RNeasy Mini Kit	Qiagen	Cat# 74104
Direct-zol RNA Miniprep Kits	Zymo Research Corporation	Cat # R2053
TRIzol™ Reagent	ThermoFisher	Cat# 15596018
Paraformaldehyde 16% Aqueous Solution EM Grade	Electron Microscopy Sciences	Cat# 15710
Donkey anti-Rabbit IgG (H+L) Highly Cross-Adsorbed Secondary Antibody, Alexa Fluor 594	ThermoFisher	A-21207
Donkey anti-Rabbit IgG (H+L) Highly Cross-Adsorbed Secondary Antibody, Alexa Fluor 647	ThermoFisher	A-31573
Donkey anti-Mouse IgG (H+L) Highly Cross-Adsorbed Secondary Antibody, Alexa Fluor 488	ThermoFisher	A-21202
Donkey anti-Mouse IgG (H+L) Highly Cross-Adsorbed Secondary Antibody, Alexa Fluor 594	ThermoFisher	A-21203
Donkey anti-Mouse IgG (H+L)	ThermoFisher	A-31571

Highly Cross-Adsorbed Secondary Antibody, Alexa Fluor 647		
Donkey anti-Rabbit IgG (H+L) Secondary Antibody, Alexa Fluor 488, Invitrogen	ThermoFisher	A-21206
Donkey anti-Goat IgG (H+L) Secondary Antibody, Alexa Fluor 594, Invitrogen	ThermoFisher	A-11058
Donkey anti Rat IgG (H+L) Highly Cross-Adsorbed Secondary Antibody, Alexa Fluor 488	ThermoFisher	A-21208
Goat anti-Guinea Pig IgG (H+L) Highly Cross-Adsorbed Secondary Antibody, Alexa Fluor™ 647	ThermoFisher	A-21450
Lectin PNA From *Arachis hypogaea* (peanut), Alexa Fluor™ 594 Conjugate	ThermoFisher	Cat# L32459
Hoechst 33342	ThermoFisher	Cat# 62249
Paraformaldehyde 16% Aqueous Solution EM Grade	Electron Microscopy Sciences	Cat# 15710
ProLong Gold antifade mountant with DAPI	Invitrogen	Cat# P36930
Propidium Iodide	Sigma Aldrich	Cat# P4170
RPMI-1640	Gibco	Cat# 11875093
DMEM, high glucose	Gibco	Cat# 11965092
Fetal Bovine Serum (FBS)	Gibco	Cat# 26140079
AGS	ATCC	CRL-1739
LS-174T	ATCC	CL-188
HEK-293T	ATCC	CRL-1573
Primocin	InvivoGen	Cat# ant-pm-1,2
Corning® 100 mL Penicillin-Streptomycin Solution, 100x	Corning	Cat# 30-002-CI
trypLE™ Express	Gibco	Cat# 12605028
RNaseOUT™ Recombinant Ribonuclease Inhibitor	ThermoFisher	Cat# 10777019
14 mL, Sterile + Certified Free	Beckman Coulter	Cat# C14302

Open-Top Thinwall Polypropylene Tube, 14 x 95mm		
Sucrose	Sigma	Cat# S0389
Isoflurane	Covetrus	Cat# 11695067772
T-PER Tissue Protein Extraction Reagent	ThermoFisher	Cat# 78510
Formaldehyde solution	Sigma-Aldrich	Cat# 252549
Histo-Clear	National Diagnostics	Cat# HS-200
DAB Substrate Kit	ThermoFisher	Cat# 36000
Vectastain Elite ABC HRP Kit	Vector Laboratories	Cat# PK-6100
Permount Mounting Medium	ThermoFisher	Cat# SP15-100
Opti-MEM™ I Reduced Serum Medium	Gibco	Cat# 31985070
Lipofectamine™ 2000 Transfection Reagent	Invitrogen™	Cat# 11668019
ChemiDoc™ MP Imaging System	Bio-Rad	Cat# 12003154
Olympus IX83 Motorized Inverted Microscope	Olympus	
Odyssey Fc Blot Imaging System	LI-COR	
Pannoramic MIDI II	Epredia	
DM6B Upright Fluorescent Microscope	Leica	
BioTek Synergy 2 Microplate Reader	BioTek	
AX R Confocal System with Eclipse Ti2-E Inverted Microscope	Nikon	
ImageJ	NIH	https://imagej.net/
Adobe Illustrator 2025	Adobe	
Photoshop 2025	Adobe	
SW 41 Ti Swining-Bucket Rotor	Beckman Coulter	Cat# 331362
Beckman Optima LE-80K Ultracentrifuge	Beckman Coulter	Cat# 365668
Piston Gradient Fractionator	BioComp	
GILSON Fraction Collection	Gilson	FC-203B

System		
Prism 10	GraphPad	https://www. graphpad.com/scientific-software/prism/
PANTHER		https://pantherdb.org/
