# Supplementary figures and images for: Stress-Responsive Protein IFRD1 Protects Assembled Ribosomes via a Ribosome-Salvaging Mechanism
Source: bioRxiv. 2026 May 7:2026.05.03.720925. Preprint. [Version 1] doi: 10.64898/2026.05.03.720925 (PMC13174501; doi:10.64898/2026.05.03.720925)

# Ribosome Salvaging

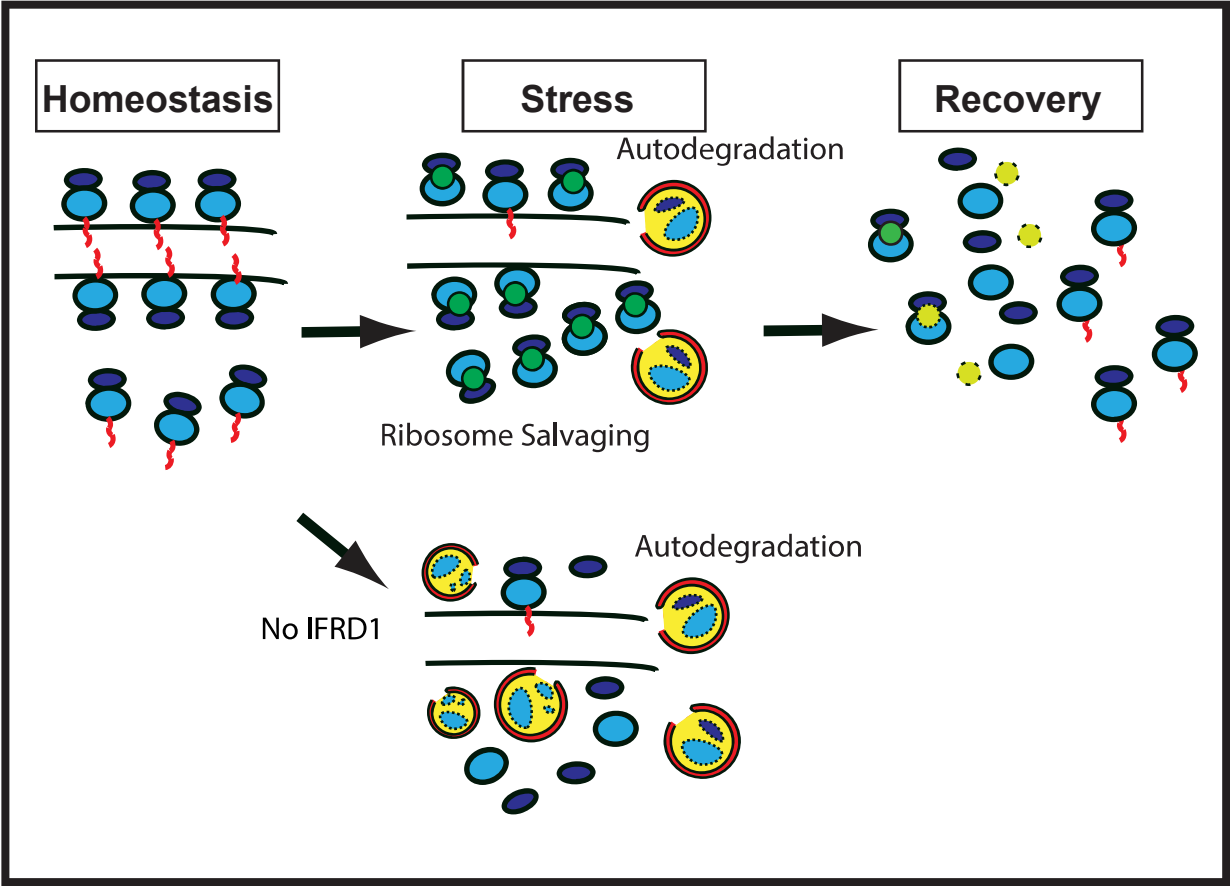

Translating Ribosome      40S Small Subunit

IFRD1      60S Large Subunit

Supplement: Supplement 4 [file media-4.pdf]
